# Prognostic Value of Tumor Markers in Gastric Cancer: A Tertiary Cancer Centre Experience

**DOI:** 10.7759/cureus.42328

**Published:** 2023-07-23

**Authors:** Pratham Batra, Arun H Narasannaiah, Venkatesh Reddy, Vignesh Subramaniyan, Manjunath K V, Yeshwanth R, Ravi Arjunan, Syed Althaf, Srinivas Chunduri, Ali Z Anwar

**Affiliations:** 1 Surgical Oncology, Kidwai Memorial Institute of Oncology, Bengaluru, IND

**Keywords:** gastric adenocarcinoma, carcinoembryonic antigen, cancer-related mortality, prognosis, ca19-9, cea, tumor marker, gastric cancer

## Abstract

Objectives: Gastric cancer is a heterogeneous malignancy in terms of stage-wise prognosis. This study aimed at finding any prognostic significance of preoperative carcinoembryonic antigen (CEA) and cancer antigen (CA) 19-9 in resectable gastric cancer.

Methods: A total of 57 patients at Kidwai Memorial Institute of Oncology, Bengaluru, India from January 2022 to March 2023 were included in this observational prospective study. Included patients had a resectable tumor at clinical staging. Patients were divided into two categories (raised and non-raised) based on serum tumor marker (CEA and CA 19-9) levels. Their relationship with clinicopathological features was studied. The association was studied using chi-square test, and p-value <0.05 was considered significant.

Results: The mean age of the study group was 55.47 years with male predominance (63.2%, n=36). Raised CEA and CA 19-9 were seen in 15.8% (n=9) and 10.5% (n=6) patients, respectively, while both markers were raised in 5.3% (n=3). Raised CEA was found significantly associated with grade 3 adenocarcinoma stomach (OR 7.825, 95%CI: 1.374-44.562; p= 0.020) and intraoperative finding of inoperability due to occult intra-abdominal disease (p<0.05). CA 19-9 (pre- and post-operative levels) had no statistically significant association (p>0.05) with the grade of adenocarcinoma.

Conclusion: This study indicates a benefit in estimating CEA for the prediction of prognosis in gastric cancer. CEA levels have been found to predict chances of finding occult intra-abdominal metastasis in gastric cancer.

## Introduction

GLOBOCAN (Global Cancer) Statistics 2020 has reported gastric cancer as the fifth most common cancer and the fourth leading cause of cancer-related mortality globally in 2020 and that the annual burden of gastric cancer is predicted to increase to 1.8 million new cases and 1.3 million deaths by 2040 [1]. TNM (tumor, node, metastasis) staging is considered the reliable standard prognosticator by the American Joint Committee on Cancer (AJCC) for the prognostication of cancer and it serves as a good guide for the treatment in gastric cancer patients [2]. Nevertheless, due to the differences in clinical and biological characteristics, the survival of gastric cancer patients with the same stage is variable, which highlights the need to look into other significant prognostic factors, such as tumor markers, which may allow the assessment of the individual prognosis of patients [2,3]. In clinical practice, cancer antigen (CA) 19-9 and carcinoembryonic antigen (CEA) are the frequently used markers for the early diagnosis and monitoring of gastric cancer [4,5]. Hence, this study attempts to find any association between the pre-operative levels of CEA and CA 19-9 with the clinicopathological features in gastric adenocarcinoma (AD) patients.

## Materials and methods

Between January 2022 to March 2023, 57 patients diagnosed with radiologically resectable, histopathologically proven gastric cancer and operated on at Kidwai Memorial Institute of Oncology, Bengaluru, India, were included in the study. The study was approved by the Medical Ethics Committee, Kidwai Memorial Institute of Oncology (approval number: KMIO/MEC/004/11.April.2022). The preoperative evaluation included complete history, thorough physical examination, routine biochemical evaluation, upper gastrointestinal endoscopy with biopsy, and staging contrast-enhanced computed tomography (CECT) of the thorax, abdomen, and pelvis. Patients with metastatic disease found on the routine preoperative evaluation protocol of our department were excluded. Patients receiving neoadjuvant chemotherapy were not included in this study. However, adjuvant chemotherapy was given to all the patients who were deemed fit after surgery. Standard surgical treatment was radical total or distal gastrectomy with D2 lymph node dissection as per the Japanese Gastric Cancer Association guidelines [6]. In selected radiologically operable cases, which were found inoperable due to involved para-aortic node or occult peritoneal disease on surgery, feeding procedures such as gastrojejunostomy or feeding jejunostomy were performed.

Serum CEA and CA 19-9 were evaluated one week preoperatively and on a second visit between one and two months postoperatively. The cut-off for CEA was considered 5 ng/ml and for CA 19-9, it was 37 U/ml. Patients were divided into two categories, namely raised and non-raised groups, where raised group included patients with either one or both markers elevated. The clinicopathological features were correlated between the two categories.

Statistical analysis

Data were entered in Microsoft Excel (Microsoft Corporation, Redmond, Washington, United States) and analyzed using IBM SPSS Statistics for Windows, Version 23.0 (Released 2015; IBM Corp., Armonk, New York, United States). Differences between groups were evaluated using Chi-square test for categorical variables. Odds ratio along with 95% confidence interval for raised marker values was calculated using logistic regression. p-value <0.05 was taken as statistically significant.

## Results

The present study included 57 patients. The mean and median age of the study group was 55.5 and 56 years, respectively, and ranged from 29 to 76 years. The majority of the study patients were male (63.2%, n=36). The cut-off for age is taken as 55 years in the present study as in a study by Lin et al., younger age was significantly associated with raised levels of tumor markers [2]. Grade 2 moderately-differentiated intestinal adenocarcinoma was found to be most prevalent (56.1%, n=32) in the present study population and grade 1 well-differentiated intestinal AD was least present. Only nine (15.8%) and six (10.5%) patients had increased pre-operative values of CEA and CA 19-9, respectively. Only three (5.3%) patients exhibited an increase in serum levels of both markers. The distal part of the stomach was found to be most commonly (n=50, 87.7%) involved in the present study population, while proximal stomach involvement was presented in only two (3.5%) patients. Three-fourths (n=43, 75.4%) of the patients underwent radical gastrectomy and 14 (24.6%) patients were found to be inoperable and underwent feeding procedures. The majority of the patients had reported T3 (n=27, 47.4%) while N1 and N2 (n=21, 36.8%) were found to be equally present. Only two patients had shown a decrease in the post-operative levels of CEA, while post-operative CA 19-9 was found to be reduced in only one patient (Table 1).

**Table 1:** The frequency distribution of various parameters in the study population. T: tumor; N: node; M: metastasis; AD: adenocarcinoma; CEA: carcinoembryonic antigen; CA: cancer antigen

		Frequency	Percentage (%)
Age	<=55	25	43.9
	>55	32	56.1
Sex	Female	21	36.8
	Male	36	63.2
T stage	1	0	0
	2	11	19.3
	3	27	47.4
	4A	16	28.1
	4B	3	5.3
N stage	0	3	5.3
	1	21	36.8
	2	21	36.8
	3	4	7.0
	3A	7	12.3
	3B	1	1.8
M stage	0	44	77.2
	1	13	22.8
Grade	AD-1	5	8.8
	AD-2	32	56.1
	AD-3	20	35.1
Location	Body	5	8.8
	Distal	50	87.7
	Proximal	2	3.5
Pre-CEA	Normal	48	84.2
	Increased	9	15.8
Pre-CA 19-9	Normal	51	89.5
	Increased	6	10.5
Operability	In- Operable	14	24.6
	Operable	43	75.4

**Figure 1:**
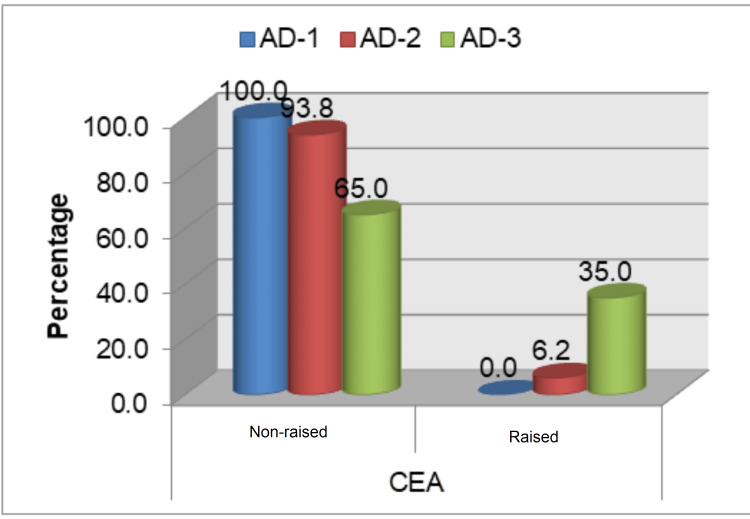
Preoperative levels of CEA with grade of adenocarcinoma AD: adenocarcinoma; CEA: carcinoembryonic antigen

**Figure 2:**
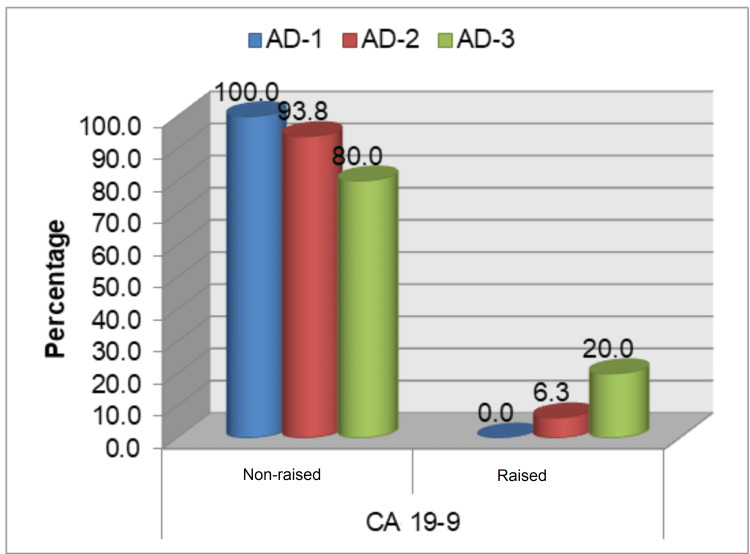
Preoperative levels of CA 19-9 with grade of adenocarcinoma AD: adenocarcinoma; CA: cancer antigen

**Figure 3:**
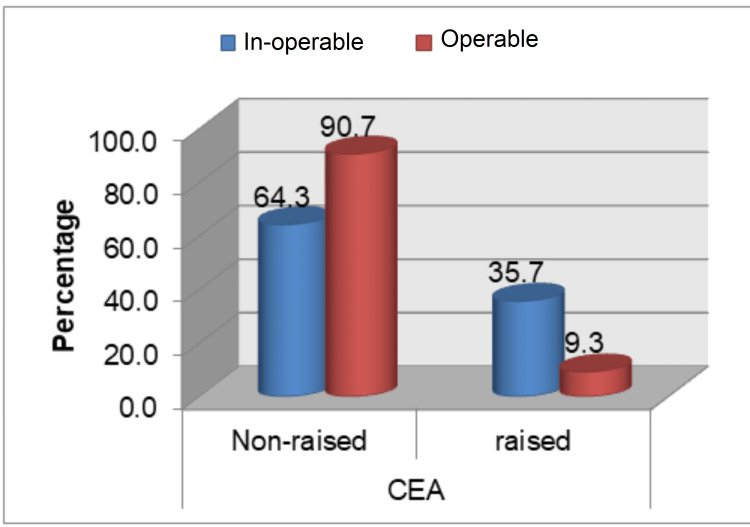
Preoperative levels of CEA with operability CEA: carcinoembryonic antigen

**Figure 4:**
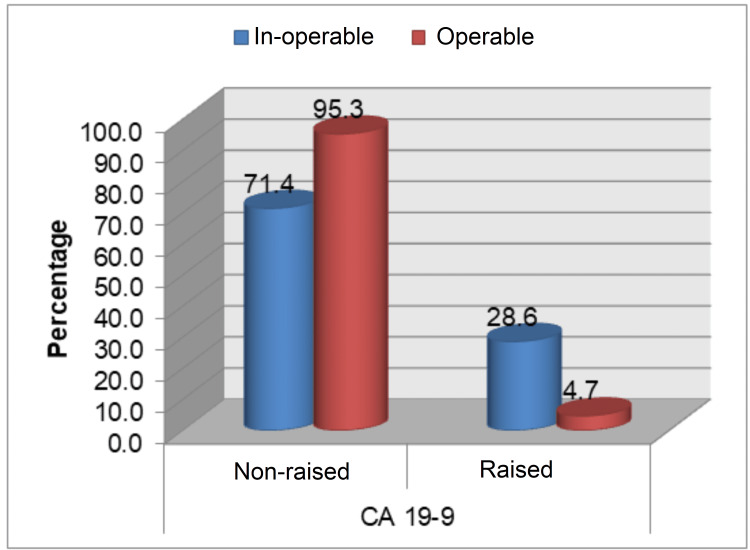
Preoperative levels of CA 19-9 with operability CA: cancer antigen

This study population did not yield any significant association between raised tumor markers (CEA and CA 19-9) pre- and post-operatively with the age of more than 55 years and gender of the study population. However, a significant association (p<0.05) was noted between grade of AD with pre- and post-operative levels of CEA, where 35% (n=7) and 30% (n=6) of total grade 3 AD patients (n=20) had increased preoperative and post-operative CEA levels, respectively (Table 2, Figure 1). No statistically significant association of the grade of AD with pre- and post-operative levels of CA 19-9 was found (Table 2, Figure 2). A statistically significant association was observed regarding the operability of cases and raised pre- and post-operative levels of CEA and CA 19-9 (Table 2, Figure 3-4). Only three cases in the present study population demonstrated an increase in the levels of both markers and they were found to have grade 3 AD and two of them were inoperable.

**Table 2:** Association of various parameters with preoperative levels of CEA and CA 19-9 * statistically significant CEA: carcinoembryonic antigen; CA: cancer antigen; T: tumor; N: node; M: metastasis

			CEA			CA 19-9		
			Non-raised	Raised	p-value	Non-raised	Raised	p-value
Age	<=55	n	23	2	.154	23	2	.583
		%	92.0%	8.0%		92.0%	8.0%	
	>55	n	25	7		28	4	
		%	78.1%	21.9%		87.5%	12.5%	
Sex	F	n	19	2	.322	20	1	.279
		%	90.5%	9.5%		95.2%	4.8%	
	M	n	29	7		31	5	
		%	80.6%	19.4%		86.1%	13.9%	
T	1	n	0	0	0.865	0	0	0.567
		%	0%	0%		0%	0%	
	2	n	9	2		10	1	
		%	81.8%	18.2%		90.9%	9.1%	
	3	n	23	4		25	2	
		%	85.2%	14.8%		92.6%	7.4%	
	4A	n	13	3		14	2	
		%	81.3%	18.8%		87.5%	12.5%	
	4B	n	3	0		2	1	
		%	100.0%	0%		66.7%	33.3%	
N	0	n	3	0	0.643	3	0	.110
		%	100.0%	0%		100.0%	0%	
	1	n	16	5		18	3	
		%	76.2%	23.8%		85.7%	14.3%	
	2	n	18	3		20	1	
		%	85.7%	14.3%		95.2%	4.8%	
	3	n	11	1		10	2	
		%	91.66%	8.34%		83.33%	16.67%	
M	0	n	40	4	0.011*	41	3	.093
		%	90.9%	9.1%		93.2%	6.8%	
	1	n	8	5		10	3	
		%	61.5%	38.5%		76.9%	23.1%	
intraoperative findings	In-Operable	n	9	5	0.019*	10	4	.011*
		%	64.3%	35.7%		71.4%	28.6%	
	operable	n	39	4		41	2	
		%	90.7%	9.3%		95.3%	4.7%	
Grade	AD-1	n	5	0	.013*	5	0	.211
		%	100.0%	0.0%		100.0%	0.0%	
	AD-2	n	30	2		30	2	
		%	93.8%	6.3%		93.8%	6.3%	
	AD-3	n	13	7		16	4	
		%	65.0%	35.0%		80.0%	20.0%	

No significant association was noted among preoperative and postoperative values of CEA and CA 19.9 with T- and N-stage of tumors; however, a statistically significant association of M was observed with pre- and post-operative levels of CEA only. two out of three patients with raised levels of both markers preoperatively had M1 stage.

Odds ratio for raised CEA and CA 19-9 with inoperability was found to be 3.807 (95%CI: 0.758-19.117; p = 0.104) and 5.599 (95%CI: 0.805-38.953; p = 0.082), respectively. Both were found to be statistically insignificant.

Odds ratio of raised CEA with grade 3 AD was found to be 7.825 (95%CI: 1.374-44.562; p = 0.020), which was statistically significant; however, for CA 19-9, it was statistically non-significant (OR = 2.475 (95%CI: 0.331-18.510); p = 0.377).

## Discussion

Gastric cancer, being the fifth most common malignancy worldwide, remains a major global health issue [1]. However, the survival of gastric cancer patients with the same stage is heterogenous despite treatment with curative intent due to variable biology. Hence, tumor markers might hold promise for further prognostication if incorporated into TNM staging. Serum CEA and CA 19-9 are frequently used markers for gastrointestinal malignancies. However, their importance remains controversial in determining gastric cancer prognosis as some studies reported its utility while others were not able to find the same [7-10]. Another reason for its limited utility is the non-specificity of these tumor markers and low positivity rates [11,12]. Therefore our study focussed on finding a correlation between raised preoperative serum CEA and/or CA 19-9 and clinic-pathological features in gastric cancer. 

The rate of preoperative raised serum CEA and CA 19-9 was 15.8% and 10.5%, respectively, while both markers were raised in 5.3% of patients in our series, similar to rates reported in other studies [2]. These markers are hypothesized to be produced by tumor cells and their serum values depend ultimately on the volume of the disease and metastasis [13,14].

There exists controversy regarding the relationship between tumor markers and clinical features of gastric cancer. The association of raised tumor markers correlated significantly with depth of invasion, TNM stage, and lymph node metastasis in some studies [15,16]. However, tumor markers were not correlated with T and N-stage in a study by Erdal Polat et al [17]. In our study, the raised markers did not correlate significantly with older age, gender, T and N staging, and location of the tumor. The plausible explanation for this could be the small sample size in our study. Even after various reports suggest the utility of tumor markers to predict TNM, stage, disease progression, and prognosis, there is no agreement as to what dynamics of change are likely to be significant.

Again there is controversy in defining the relation of tumor markers with histological grade where some authors found a low positivity rate of CEA, CA 19-9, and CA 72-4 in diffuse-type gastric cancer while others found vice versa [18,19]. Our study showed a significant association between raised CEA values and histopathological grade where grade 3 patients were found to have higher values. This association was not seen with CA 19-9, which again could possibly be because of less number of patients. As we know that high grade is associated with poor prognosis, our study indicates that raised markers suggest poor prognosis in gastric cancer patients.

There are some studies that suggest the prognostic role of postoperative values of CEA and CA 19-9 where they found raised markers to be associated with worse overall survival [20]. In our study, postoperative markers were reduced in only three patients postoperatively but a longer duration of follow-up is required to assess the survival outcomes.

Gastric cancer is known to have occult intra-abdominal metastatic disease, which cannot be reliably confirmed preoperatively despite modern imaging [21]. In our study too, around 25% of patients were found to be inoperable due to either para-aortic lymph node, peritoneal, or liver metastasis, which was significantly correlated with raised tumor markers. These findings indicate that serum marker values increase with the progression of the disease.

There are certain limitations of this study. Firstly, the study is limited by the small number of patients, and therefore further stratified analysis cannot be carried out. Secondly, the duration of follow-up is short; hence, the correlation with overall survival and disease-free interval cannot be measured. Lastly, with the rapid development of liquid biopsies and molecular genetics, there may be a role of other markers in the prognostication of gastric cancer.

## Conclusions

Raised preoperative serum CEA is a valuable predictor of high tumor grade in gastric cancer. This also provides additional information to that obtained by conventional staging methods regarding occult metastasis. Although further research is warranted with a larger sample size, serum CEA may be considered as an independent prognostic factor in clinically operable gastric cancer patients.
